# Changes in Sugar-Sweetened Beverage Intake From Before to After Nutrition Labeling Policy Implementation: A Comparison of Mexico and the United States

**DOI:** 10.5888/pcd23.250422

**Published:** 2026-06-11

**Authors:** Alejandra Contreras-Manzano, Sultan Alsallum, Rachel E. Davis, Lana Vanderlee, Alejandra Jáuregui, Christine M. White, Virginie Drolet-Labelle, Edward A. Frongillo, Laura Lázaro, Simón Barquera, Lindsey Smith Taillie, James W. Hardin, David Hammond, James F. Thrasher

**Affiliations:** 1 Center for Research in Nutrition and Health (CINyS), Instituto Nacional de Salud Pública, Cuernavaca, Mexico; 2 National Council for Humanities, Science, and Technology, Mexico City, Mexico; 3 Department of Epidemiology and Biostatistics, Arnold School of Public Health, University of South Carolina, Columbia, South Carolina; 4 Department of Health Promotion, Education and Behavior, Arnold School of Public Health, University of South Carolina, Columbia, South Carolina; 5 Le Centre Nutrition, Santé et Société (NUTRISS), École de Nutrition de l’Université Laval, Quebec City, Quebec, Canada; 6 School of Public Health Sciences, University of Waterloo, Waterloo, Ontario, Canada; 7 Department of Nutrition, Gillings School of Global Public Health, and Carolina Population Center, University of North Carolina at Chapel Hill, Chapel Hill, North Carolina

## Abstract

**Introduction:**

Sugar-sweetened beverages (SSBs) are a major source of added dietary sugars in Mexico and the US and contribute to chronic disease risk. In 2020, Mexico implemented mandatory front-of-package warning labels (FoPWLs), and the US implemented updated Nutrition Facts labels that increased the salience of calories and added sugars. We compared changes in SSB intake in Mexico and the US.

**Methods:**

Using 6 annual cross-sectional surveys (2018–2023) from the International Food Policy Study, we analyzed Mexican (n = 24,574) and US (n = 25,019) adults. Past-week SSB intake was summarized across 5 SSB categories. Outcomes were any SSB consumption, SSB consumption frequency, and, among SSB consumers, total volume, calories, and added sugars from SSBs. Difference-in-differences models with country-by-period interactions (2018–2019 vs 2020–2023) adjusted for sociodemographic and behavioral covariates; sex-stratified analyses were also conducted.

**Results:**

From 2018–2019 to 2020–2023, any SSB consumption and SSB consumption frequency increased in the US (adjusted odds ratio, 1.26; 95% CI, 1.17–1.36; adjusted incidence rate ratio, 1.16; 95% CI, 1.10–1.22, respectively). In Mexico, any SSB consumption also increased (adjusted odds ratio, 1.11; 95% CI, 1.00–1.22), but increases were smaller than in the US for any consumption (adjusted odds ratio, 0.88; 95% CI, 0.77–0.99) and frequency (adjusted incidence rate ratio, 0.89; 95% CI, 0.84–0.95). Among SSB consumers, changes in volume, calories, and added sugars did not differ between countries overall. In sex-stratified models, Mexican women had a greater relative reduction in SSB volume than US women (adjusted regression coefficient, −304 mL/week; 95% CI, −593 to −16).

**Conclusion:**

Compared with contemporaneous changes in the US, Mexico’s FoPWL implementation was associated with more favorable trends in whether and how often adults consumed SSBs, but not in overall changes in volume, calories, or added sugars among SSB consumers.

**Summary**

**What is already known on this topic?**

Front-of-package warning labels (FoPWLs), implemented in Mexico in 2020, may reduce sugar-sweetened beverage (SSB) consumption, but population-based evidence on changes in SSB intake after implementation remains limited.

**What is added by this report?**

Using a difference-in-differences design, we found that from 2018 through 2019 to 2020 through 2023, increases in any past-week SSB consumption and SSB consumption frequency were smaller in Mexico than in the US, whereas changes in volume, calories, and added sugars among SSB consumers were broadly similar.

**What are the implications for public health practice?**

FoPWLs may help curb whether and how often adults consume SSBs. Additional measures may be needed to reduce the amount of SSBs consumed and the calories and added sugars consumed among people who already drink them.

## Introduction

Worldwide, noncommunicable diseases accounted for 18 million premature deaths in 2021 (1); sugar-sweetened beverage (SSB) intake accounted for approximately 2.2 million new cases of type 2 diabetes and 1.2 million new cases of cardiovascular disease in 2020, with the largest proportional burdens in Latin America and the Caribbean (2). Biological pathways through which SSBs affect health include high-glycemic glucose loads, fructose-driven hepatic lipogenesis, weight gain, and downstream dyslipidemia, hypertension, and systemic inflammation (3). SSBs comprise the largest single source of added sugars in both the US (4) and Mexico (5). Approximately 93% of Mexican adults reported consuming an SSB the previous day in 2020 through 2024 (6). About two-thirds (63%) of US adults drink SSBs at least once daily (4). As of 2024–2025, annual per-capita soda volumes were approximately 163 liters in Mexico and 118 liters in the US, placing both countries among the world’s highest consumers of these beverages (7,8).

Mexico has implemented various policies to influence SSB purchasing and consumption. A 2014 excise tax of 1.0 Mexican peso per liter on SSBs was associated with reduced taxed beverage purchases, especially among lower-income groups (9). In 2020, Mexico implemented front-of-package warning labels (FoPWLs) to identify products with high amounts of calories, added sugars, saturated fats, trans fats, or sodium, according to the Pan American Health Organization nutrient profile (10). Octagonal, black and white “stop sign” FoPWLs indicate “EXCESS” amounts for each characteristic, with less healthy products potentially displaying all 5 FoPWLs. Chile first implemented FoPWLs in 2016, and evidence of their beneficial effects spurred FoPWL adoption in Mexico and, subsequently, in other Latin American countries (11,12). After Mexico’s FoPWL implementation in 2020, almost half of Mexican adults reported reducing SSB purchases, and among those who reported greater reductions in purchases, they also reported consuming fewer SSBs in the prior week (13). However, pre–post data on consumption are lacking.

Our study assessed changes in SSB intake after Mexico’s implementation of FoPWLs, using the US as a comparison setting because it did not implement mandatory FoPWLs during the study period but did implement updated Nutrition Facts labels that increased the salience of calories, serving size, and added sugars (14). The same major SSB companies dominate both markets (7,8), making the cross-country comparison informative, although not equivalent to an untreated control.

## Methods

### Study design and sample

We analyzed 2018 through 2023 data from the Mexico and US arms of the International Food Policy Study (IFPS) (15), an annual cross-sectional online survey among adults in 6 countries (US n = 25,019; Mexico n = 24,574). Participants were recruited from nonprobability online consumer panels from the Nielsen Consumer Insights Global Panel and their partners. Email and panelist dashboard app invitations were sent to random samples of panelists after targeting by age and sex to help meet quotas that approximated national population distributions. Response rates were lowest in 2023 (Mexico, 2.8%; US, 1.6%) and highest in 2021 (Mexico, 16.2%; US, 12.0%). Among eligible people who accessed the survey link, around half or more completed the survey in Mexico (2018, 71.8%; 2019, 60.1%; 2020, 49.8%; 2021, 67.7%; 2022, 64.6%; 2023, 64.0%) and the US (2018, 68.0%; 2019, 49.9%; 2020, 63.3%; 2021, 69.2%; 2022, 67.9%; 2023, 66.4%). Poststratification survey weights to help correct for potential selection and sampling bias used demographic benchmarks from national census data on age, sex at birth, region of residence, education (US only), and ethnicity. Participants provided informed consent after screening and before starting the survey, which was self-administered in English or Spanish in the US, and in Spanish in Mexico, with standard panel incentives for participation (eg, points-based rewards). The study received ethics approval through a University of Waterloo Research Ethics Board (REB# 30829) in Canada and collaborating institutions in Mexico and the US.

### Measures

#### Outcomes

The Beverage Frequency Questionnaire (BFQ) captured participants’ past-week consumption of 24 beverage types (16). Participants reported the number of times they consumed each beverage type over the prior 7 days, with follow-up questions on their typical portion size consumed, showing images of container types with their associated volume. We limited the beverage types analyzed to SSBs that were likely to display FoPWLs (17) based on nutrition composition (18,19) and market assessments: regular soda, sweetened fruit drinks (eg, fruit punch or fruit nectars with added sugars), flavored or vitamin-enhanced waters, regular sports drinks, and regular energy drinks. The BFQ has been validated against 7-day food records among Canadians aged 16 to 30 years, with each beverage category in the current study having moderate to high correlations for consumption frequency (range, 0.46–0.91) and total volume (range, 0.48–0.95), as well as for aggregate SSBs (0.53 and 0.54, respectively) and sugary drinks (0.63 and 0.55, respectively) (16). For sensitivity analyses, we added 100% fruit and vegetable juice consumption to the SSB measure because some consumers confuse these with sweetened fruit drinks, although FoPWLs were not mandated for these drinks because of the FoPWL policy’s focus on added sugars.

For each beverage type and for a composite measure combining all SSB categories, we derived 5 outcomes: any consumption in the prior 7 days (yes, no); consumption frequency (number of times consumed in the prior 7 days); and, among consumers only, total volume consumed (frequency multiplied by typical portion size, in milliliters), calories consumed, and added sugars consumed. We excluded participants with no SSB consumption from models of volume, calories, and added sugars because including zero values produced highly skewed distributions and poor model fit. To calculate calories and added sugars, we used country-specific conversion factors based on market share by BFQ beverage category and corresponding nutrient data. For the US, we used nutrient composition data from the Nielsen IQ Homescan panel (2015–2018) (18), which links Nutrition Facts label information from packaged beverages with household purchases. These values were weighted by using nationally representative household projection factors to reflect actual market share. For Mexico, we updated nutrient composition data used for the National Survey on Health and Nutrition (ENSANUT 2012) by using nutrition facts panel information from beverages collected in the Mexican market for both prepolicy and postpolicy periods (2016–2018 and 2020–2023, respectively), according to A. Contreras-Manzano, DSc, (unpublished chapter manuscript, April 2026). The databases include 663 beverage products from 2016 through 2018 and 554 from 2023 through 2024, with 333 products present in both periods. In both countries, beverage categories for nutrient composition analysis were aligned with the BFQ.

#### Covariates

We adjusted for sociodemographic and behavioral covariates relevant to beverage consumption and potential to confound focal associations of interest (13). Participants self-reported their sex at birth (male, female), age (recoded: 18–29, 30–44, 45–59, 60 years or older), and education (recoded: low = high school diploma or less; medium = some college or technical training; high = university degree or higher). Perceived income adequacy was measured by asking participants to report how easy it has been to make ends meet (1 = very difficult to 5 = very easy) (20). Body mass index was classified as less than 18.5, 18.5 to 24.9, 25 to 29.9, and 30 or higher on the basis of self-reported height and weight (21). We maintained a missing category for BMI for people who answered “don’t know” or “refused” for these outcomes or who reported extreme values (13.1%). The number of children aged younger than 18 years living in the household was recategorized as none, 1, or 2 or more, as attention to nutrition labels — some of which focus on children’s health — may vary as a function of having children living in the home. We also included household shopping responsibility as a binary indicator (primary vs nonprimary shopper) to reflect opportunities for label exposure when purchasing foods.

### Statistical analysis

Missing data resulted primarily from incomplete responses for SSB measures (10.0%; n = 4,938), whereas 1.4% (n = 732) had missing data for at least 1 covariate. We applied a difference-in-differences (DiD) design to assess changes in SSB consumption outcomes over time in Mexico relative to the US. The 2 primary independent variables were country (US [reference] vs Mexico) and policy period (prepolicy, 2018–2019 [reference], vs postpolicy, 2020–2023). The modeling strategy depended on outcome characteristics. For the binary outcome of any versus no consumption, we estimated logistic regression models. For consumption frequency outcomes, we estimated negative binomial regression models because the data were counts and included zero values. For volume outcomes, as well as intake of calories and added sugars, we estimated linear regression models after excluding participants who reported no consumption of the beverage(s) of interest because inclusion of zero values violated distributional assumptions. Our DiD analysis pooled data from both countries, coding surveys as prepolicy (2018–2019) or postpolicy (2020–2023) to estimate within-country changes from prepolicy to postpolicy; the country-by-period interaction estimated the difference in changes over time in Mexico relative to the US. To assess the parallel-trends assumption, we limited data to the 2018 and 2019 surveys and tested interactions between prepolicy time and country; results indicated no difference in trends during the prepolicy period (Wald χ^2^ [df = 1] range, 0.31–1.52; *P* value range, .22–.58).

All models adjusted for key covariates and used poststratification weights rescaled to the analytic sample size. We report adjusted odds ratios (aORs), adjusted incidence rate ratios (aIRRs), and adjusted regression coefficients (β*_adj_*), as appropriate, with 95% CIs. Model diagnostics, including checks for multicollinearity and overall model fit, indicated no major concerns (eg, VIF <1.3). Postestimation commands were used to generate adjusted yearly estimates for each country. We also estimated sex interaction models and sex-stratified models. Sensitivity analyses incorporated 100% fruit or vegetable juice into a sugary-drinks measure, excluded 2020 data, and used multiple imputation with chained equations, generating 20 imputed data sets with estimates pooled using Rubin’s rules. Analyses were conducted in SAS version 9.4 (SAS Institute).

## Results

### Sample characteristics

In both countries, the weighted samples were approximately evenly divided by sex, but the countries differed across all measured characteristics (Table 1). High educational attainment was more common in Mexico than in the US, and the US sample was older overall. The Figure shows adjusted yearly estimates of any SSB consumption, SSB consumption frequency, and, among SSB consumers, volume, calories, and added sugars.

**Table 1:** Weighted and Unweighted Characteristics of the Mexican and US Samples, 2018–2023

Variable	Mexico (n = 24,574)		US (n = 25,019)		*P* value^a^
	Unweighted %	Weighted %	Unweighted %	Weighted %	
**Sex**					
Male	50.9	47.9	48.5	48.9	.06
Female	49.0	52.1	51.4	51.1	
**Age group, y**					
18–29	31.8	28.6	18.3	20.1	<.001
30–44	33.8	31.4	25.5	25.4	
45–59	28.2	28.6	24.8	24.8	
≥60	6.2	11.3	31.4	29.6	
**Education^b^**					
Low	20.8	21.2	36.4	55.9	<.001
Medium	12.9	13.8	21.0	9.9	
High	66.2	65.0	42.5	34.1	
**Race and ethnicity**					
Non-Latino White	NA	NA	71.7	63.8	NA
Non-Latino Black			8.3	10.9	
Non-Latino Other			4.5	6.0	
Non-Latino Multiracial			6.3	7.3	
Latino			9.2	12.0	
**Perceived income adequacy^c^**					
Very difficult	10.9	11.9	9.0	10.1	<.001
Difficult	31.1	32.0	19.0	20.1	
Neither easy nor difficult	39.5	39.0	32.1	33.6	
Easy	14.5	13.4	23.0	21.0	
Very easy	4.0	3.7	16.9	15.2	
**Body mass index^d^**					
<18.5	2.2	2.0	2.7	2.9	<.001
18.5–24.9	38.8	37.8	32.9	32.0	
25.0–29.9	30.1	30.5	28.8	27.8	
≥30.0	14.2	14.8	24.0	24.5	
Missing	14.7	14.9	11.4	12.8	
**Children at home**					
None	46.3	49.9	70.4	71.2	<.001
1	24.6	23.3	14.4	14.0	
≥2	29.1	26.8	15.2	14.8	
**Food shopping role**					
Primary shopper	72.7	73.4	71.4	69.9	<.001
Nonprimary shopper	27.4	26.6	28.6	30.1	
**Year of survey**					
2018	16.7	16.7	18.3	18.3	<.001
2019	17.4	17.4	16.4	16.4	
2020	17.2	17.2	18.1	18.1	
2021	16.8	16.8	16.0	16.0	
2022	16.7	16.7	15.6	15.6	
2023	15.2	15.2	15.5	15.5	

Abbreviation: NA, not applicable.

a *P* value is for omnibus χ^2^ test assessing weighted Mexican and US samples.

b Education categories were defined as low (high school diploma or less), medium (some college or technical training), and high (university degree or higher).

c Participants were asked to report how easy it has been to make ends meet.

d Based on self-reported height and weight.

**Figure:**
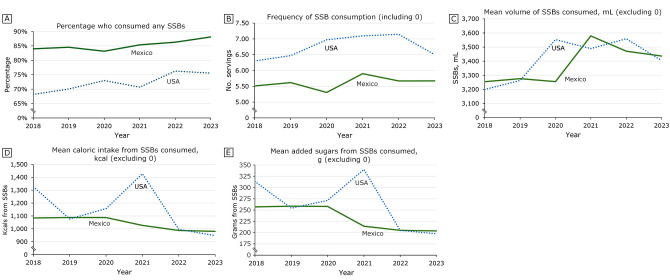
Trends in past-week sugar-sweetened beverage (SSB) intake among Mexican and US adults, 2018–2023. Estimates are adjusted for covariates and poststratification weights, and Panels A–E show, respectively, any SSB consumption, consumption frequency, and, among SSB consumers, volume, calories, and added sugars.

**Table:** 

Country	2018	2019	2020	2021	2022	2023
**Percentage who consumed any SSBs**						
Mexico	84	85	83	85	86	88
US	68	70	73	71	76	76
**Frequency of SSB consumption (including 0)**						
Mexico	5.51	5.62	5.31	5.90	5.67	5.67
US	6.30	6.47	6.97	7.09	7.15	6.50
**Mean volume of SSBs consumed (excluding 0), mL**						
Mexico	3,254	3,276	3,254	3,579	3,471	3,437
US	3,199	3,263	3,553	3,489	3,559	3,404
**Mean caloric intake from SSBs consumed (excluding 0), kcal**						
Mexico	1,084	1,088	1,087	1,026	987	980
US	1,326	1,077	1,157	1,427	996	948
**Mean added sugars from SSBs consumed (excluding 0), g**						
Mexico	257	259	258	214	205	204
US	313	254	271	340	205	197

### Changes in any SSB consumption

Any SSB consumption in the prior week (Figure, panel A; Table 2) increased from the prepolicy to postpolicy period in both Mexico (aOR, 1.11; 95% CI, 1.00–1.22) and the US (aOR, 1.26; 95% CI, 1.17–1.36). However, the increase was smaller in Mexico than in the US (aOR, 0.88; 95% CI, 0.77–0.99). In sex-stratified models, this relative difference appeared more pronounced among men (aOR, 0.78, 95% CI, 0.66–0.93), although the sex interaction was not significant (χ^2^ = 3.02; *P* = .08).

**Table 2:** Changes in Sugar-Sweetened Beverage (SSB) Intake From Before (2018–2019) to After (2020–2023) Labeling Policy Implementation Among Mexican and US Adults, 2018–2023^a^

Population	Any consumption	Consumption frequency	Volume, mL	Caloric intake, kcal	Added sugars, g
	aOR^b^ (95% CI)	aIRR^c^ (95% CI)	β*_adj_* d (95% CI)	β*_adj_* e (95% CI)	β*_adj_* f (95% CI)
**Entire population**					
US (within country)	1.26 (1.17 to 1.36)^g^	1.16 (1.10 to 1.22)^g^	272.9 (98.53 to 447.22)^h^	−70.65 (−136.10 to −5.21)^i^	−30.71 (−46.21 to −15.22)^g^
Mexico (within country)	1.11 (1.00 to 1.22)^i^	1.03 (0.99 to 1.07)	168.9 (43.54 to 294.29)^h^	−64.62 (−105.46 to −23.77)^h^	−37.16 (−46.03 to −28.28)^g^
Mexico vs US	0.88 (0.77 to 0.99)^i^	0.89 (0.84 to 0.95)^g^	−103.96 (−318.89 to 110.96)	6.03 (−71.12 to 83.19)	−6.45 (−24.72 to 11.83)
**Men only**					
US (within country)	1.31 (1.18 to 1.45)^g^	1.13 (1.06 to 1.21)^g^	86.97 (−175.90 to 349.84)	−150.21 (−248.79 to −51.63)^h^	−49.75 (−73.19 to −26.32)^g^
Mexico (within country)	1.02 (0.90 to 1.17)	0.98 (0.94 to 1.03)	175.51 (1.14 to 349.87)^i^	−76.73 (−133.47 to −19.98)^h^	−41.03 (−54.49 to −27.56)^g^
Mexico vs US	0.78 (0.66 to 0.93)^h^	0.87 (0.80 to 0.94)^g^	88.54 (−226.57 to 403.65)	73.48 (−40.03 to 187.00)	8.73 (−18.25 to 35.70)
**Women only**					
US (within country)	1.21 (1.10 to 1.34)^g^	1.18 (1.09 to 1.27)^g^	468.44 (243.09 to 693.79)^g^	13.18 (−71.39 to 97.75)	−10.54 (−30.45 to 9.37)
Mexico (within country)	1.19 (1.03 to 1.36)^i^	1.08 (1.01 to 1.15)^i^	164.26 (−15.16 to 343.69)	−52.60 (−111.08 to 5.88)	−33.32 (−47.15 to −19.49)^g^
Mexico vs US	0.98 (0.82 to 1.16)	0.92 (0.83 to 1.01)	−304.17 (−592.76 to −15.58)^i^	−65.78 (−168.53 to 36.96)	−22.78 (−47.01 to 1.44)

Abbreviations: aOR, adjusted odds ratio; aIRR, adjusted incidence rate ratio; β*_adj_*, adjusted regression coefficient.

a Adjusted covariates: age category, education level, perceived income adequacy, body mass index category, number of children in the household, household shopping responsibility, and sex.

b Results from logistic regression models for binary outcome of any beverage use in the past week vs no use.

c Results from negative binomial regression models for the number of drinks in the past week (including no drinks).

d Results from linear regression models for total volume in the past week (excluding people who did not drink any SSBs).

e Results from linear regression models for total calories intake in the past week (excluding people who did not drink any SSBs).

f Results from linear regression models for total added sugars in the past week (excluding people who did not drink any SSBs).

g Significant at *P* < .001.

h Significant at *P* < .01.

i Significant at *P* < .05.

### Changes in frequency of consuming SSBs

From the prepolicy to postpolicy period, past-week SSB consumption frequency (Figure, panel B; Table 2) increased in the US (aIRR, 1.16; 95% CI, 1.10–1.22) but not in Mexico (aIRR, 1.03; 95% CI, 0.99–1.07). The increase was smaller in Mexico than in the US (aIRR, 0.89, 95% CI, 0.84–0.95). In sex-stratified models, the relative difference was somewhat larger among men (aIRR, 0.87; 95% CI, 0.80–0.94) than women (aIRR, 0.92; 95% CI, 0.83–1.01), but the sex interaction was not significant (χ^2^ = 0.36; *P* = .55).

### Changes in volume of SSB consumption among SSB consumers

Among SSB consumers, past-week SSB volume (Figure, panel C; Table 2) increased in both Mexico (β*_adj_*, 169 mL; 95% CI, 44–294 mL) and the US (β*_adj_*, 273 mL; 95% CI, 99–447 mL), with no overall between-country difference in change over time. In sex-stratified models, Mexican women had a greater relative reduction in volume than US women (β*_adj_*, −304 mL; 95% CI, −593 to −16 mL), although the sex interaction was marginal (χ^2^ = 3.16; *P* = .08).

### Changes in intake of calories and added sugars from SSBs among SSB consumers

In Mexico, weekly caloric intake from SSBs decreased from the prepolicy period (β*_adj_*, −65 kcal; 95% CI, −105 to −24 kcal), as did added sugars intake (β*_adj_*, −37 g; 95% CI, −46 to −28 g) (Figure, panels D and E; Table 2). Similar decreases were observed in the US for calories (β*_adj_*, −71 kcal, 95% CI, −136 to −5 kcal) and for added sugars (β*_adj_*, −31 g; 95% CI, −46 to −15 g). No differences were found between countries in these prepolicy to postpolicy trends, including in sex-stratified models. Interaction models suggested marginal effect modification by sex (calories χ^2^ = 3.08; df = 1; *P* = .08; added sugars χ^2^ = 2.81; df = 1; *P* = .09).

### Sensitivity analysis

The magnitude and direction of findings were generally consistent across sensitivity analyses (available on request from corresponding author). When 100% fruit or vegetable juice was added to create a sugary-beverages measure, the overall between-country pattern remained similar: compared with the US, Mexico had smaller increases in any consumption (aOR, 0.77; 95% CI, 0.67–0.89) and consumption frequency (aIRR, 0.87; 95% CI, 0.83–0.92), with no between-country differences in volume, calories, or added sugars. Among women, the difference in consumption frequency remained (aIRR, 0.88; 95% CI, 0.81–0.96), but the difference in volume did not. After excluding 2020 data, the smaller increase in any SSB consumption in Mexico relative to the US was no longer evident, and the female-specific difference in SSB volume was no longer significant. Multiple-imputation analyses were also broadly consistent with the primary findings, although the between-country difference in consumption frequency and the female-specific volume finding were attenuated.

## Discussion

In this multiyear comparative analysis of adults in Mexico and the US, we found more favorable relative trends in Mexico for any past-week SSB consumption and SSB consumption frequency from the prepolicy to postpolicy period. By contrast, among SSB consumers, changes in weekly volume, calories, and added sugars were broadly similar across countries. These findings were robust to sensitivity analyses, although some sex-specific findings were not. The results suggest that Mexico’s mandatory FoPWLs and updated Nutrition Facts label (eg, bolded constituents highlighted on FoPWLs) attenuated increases in SSB consumption compared with the consumption in US, where Nutrition Facts labels printed on the side or back of packaging also were updated in 2020–2021 to emphasize calories and added sugars. However, prepolicy to postpolicy trends in SSB calories and added sugars were similar across countries, suggesting that Mexico’s labeling did not outperform the US for these outcomes. Finding mixed effects has also typified other studies of front-of-package labeling systems in real-world contexts (12).

Smaller increases in any SSB consumption and consumption frequency in Mexico than in the US suggest that Mexico’s labeling and broader policy environment may have influenced the extensive margin of SSB consumption (ie, whether and how often people choose these beverages) (22). In 2020–2021, for example, around 50% of Mexican adults reported decreasing their soda purchases because of FoPWLs (13). Nevertheless, we did not find absolute prepolicy to postpolicy decreases in any SSB consumption or SSB consumption frequency in Mexico. Hence, the main effects appear to stem from deflecting the positive trend in consumption that would have happened in the absence of FoPWLs, with greater increases in the US than Mexico. These positive trends are consistent with sales data on SSB consumption in Mexico, the US, and across the Americas (7,8), as well as with our data showing increasing prepolicy trends from 2018 to 2019 (Figure, panels A and B).

Among people who consumed SSBs, Mexico’s FoPWL implementation appeared less clearly related to intensive-margin outcomes (ie, how much people consumed once they chose SSBs). In both countries, weekly volume increased among consumers, whereas calories and added sugars decreased. These patterns are consistent with the possibility that reformulation contributed to lower caloric and sugar intake even when consumption frequency or volume did not decline. Because this study used repeated cross-sectional survey data and a nonequivalent comparison country, these findings should be interpreted as associations rather than causal effects of labeling alone. External sales data from Mexico provide related context: after the Mexican FoPWL policy was announced, sales volume of SSBs declined by 5% (−5.0%, mL per capita per month, *P* < .001) as did calories by 3.7% (−3.7%, per capita per month, *P* < .001) and added sugars by 38% (−38.0%, per capita per month, *P* < .001) with larger reductions after implementation and persistence in conservative analyses addressing COVID-19–related effects (23). Similar declines were observed in Chile after implementation of FoPWLs (24). Although our survey estimates of weekly SSB volume among consumers increased, the concurrent declines in calories and added sugars in Mexico may reflect product reformulation despite stable consumption frequency. Reformulation associated with FoPWLs was estimated to reduce beverage energy by 3 kcal per 100 mL (ie, around 7 g of sugar per liter) (25), which could be meaningful in a high-consumption setting such as Mexico (26). Industry reformulation in response to front-of-package labeling policies is common and can result in meaningful public health benefits (27). To our knowledge, SSB reformulation is not widespread in the US, and policy-based incentives (eg, taxes, labeling) are generally required to motivate such changes (28). Additional studies with nationally representative data should more rigorously assess population-level effects of Mexico’s FoPWL policy.

Sex differences should be interpreted cautiously. Relative improvements among men were concentrated in any SSB consumption and consumption frequency, whereas among females the only apparent relative improvement was for SSB volume among consumers, and that finding weakened in sensitivity analyses. This pattern may be consistent with prior studies suggesting that women may respond more strongly than men to FoPWLs and related health messaging, but it requires confirmation in future studies (13,29,30). Future research should examine why men, who often consume more nonalcoholic beverages, including SSBs, appeared more likely to show relative improvements in the likelihood and frequency of consumption without comparable reductions in volume (31). Socioeconomic status, age, and household composition could be explored given evidence that label comprehension and product affordability vary across these groups.

### Limitations

Our study has some limitations. Participants were recruited from nonprobability online panels. Despite efforts to recruit people whose sociodemographic profiles matched the general population and adjustment of estimates by using poststratification weights, residual selection biases may nevertheless have influenced the generalizability of our results. In particular, our Mexico sample underrepresented people with lower educational attainment, although this may have resulted in overestimates given that FoPWLs appear more effective for people with low education (13). Nevertheless, evidence is generally more mixed for education-related differences across front-of-package labeling systems (32). Furthermore, the BFQ relies on self-report that may misclassify portion sizes or fail to capture unbranded or unpackaged beverages. For example, prepolicy versions of the BFQ did not capture sweetened coffees and teas that have become increasingly popular in the US, and it did not explicitly assess *aguas frescas*, which Mexican people often prepare at home, usually with added sugars (33). Consumers in either country may have substituted these beverages for the SSBs we captured, with unknown effects on our estimates. Even with our potentially limited measurement, however, the frequency and volume of SSB consumption increased in both countries, suggesting that substitution was less likely than addition. Although the US offers a reasonable comparison with Mexico, especially given the increasingly integrated food industry across these countries, differences across our samples and unmeasured country-specific characteristics and shocks (eg, marketing, local taxes, supply changes) could have introduced bias. Moreover, because the US also implemented label changes during the study period, it should be viewed as a comparison setting rather than an untreated control group. Furthermore, many Mexican Americans have reported encountering FoPWLs in Mexican-oriented stores and reducing their SSB purchases in response (29), thereby potentially contaminating our US comparison group. Future research should evaluate differences in SSB trends by race and ethnicity subgroups, whose samples were too small for us to meaningfully assess. In addition, our design could not fully separate FoPWL effects from market dynamics in Mexico, including industry efforts to weaken regulations (11) and massive reformulation effects (25) that likely help account for some of the reductions in calorie and added sugar intake. Because of challenges with model convergence, volume-based outcomes were estimated only among SSB consumers. Given that the prepolicy to postpolicy increases in any SSB consumption were less dramatic in Mexico than the US, exclusion of non-SSB consumers likely attenuated any cross-country differences in volume outcomes.

### Conclusion

In this 6-year, 2-country comparison, Mexico showed more favorable relative trends than the US in whether and how often adults consumed SSBs after implementation of mandatory FoPWLs, but not in overall changes in volume, calories, or added sugars among SSB consumers. These findings suggest that FoPWLs, especially when implemented within a broader policy environment that includes taxation and reformulation incentives, may help curb SSB consumption decisions at the point of selection. Additional policies are likely needed to produce larger reductions in caloric and added-sugar intake among people who already consume SSBs.
